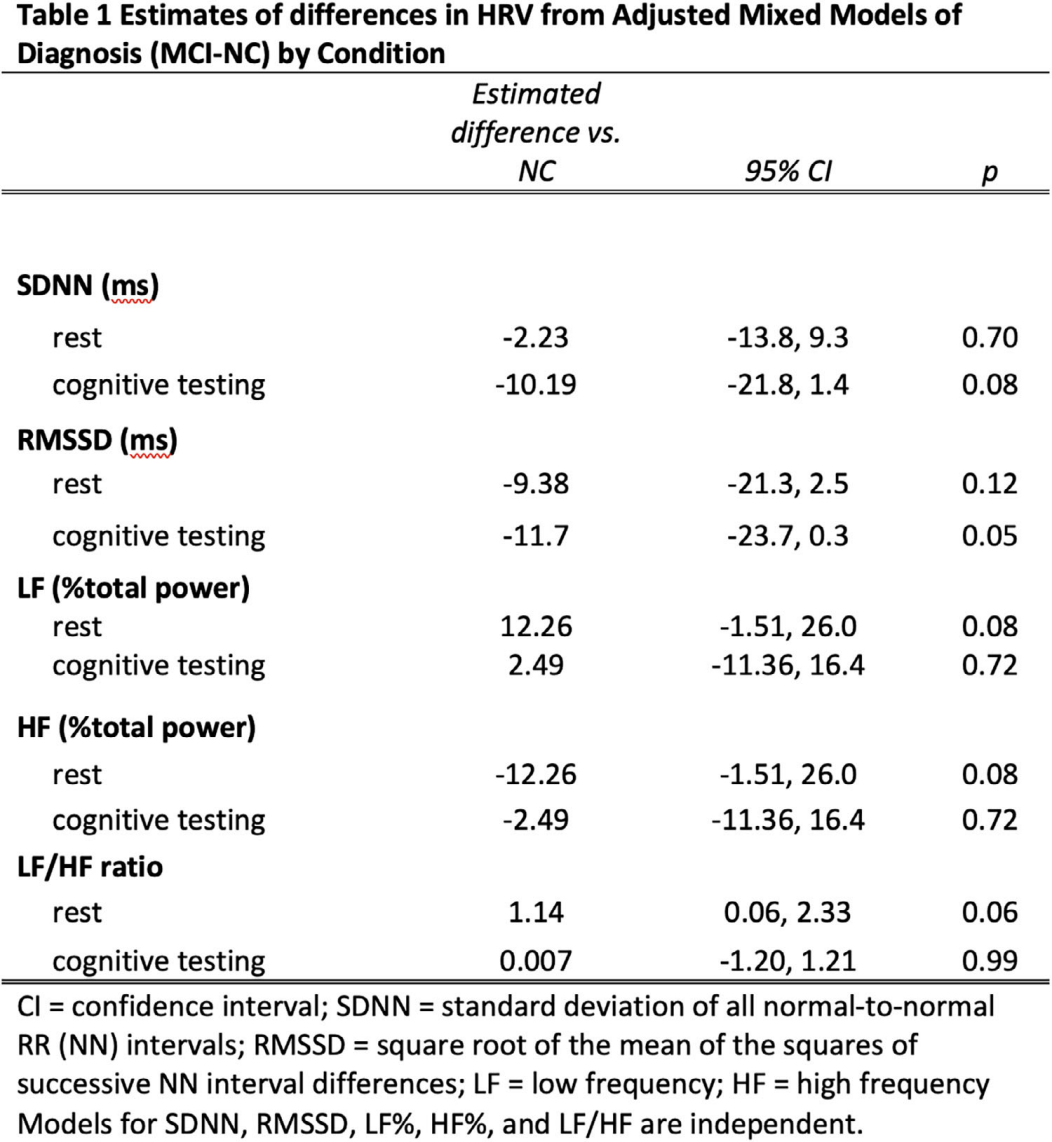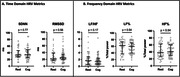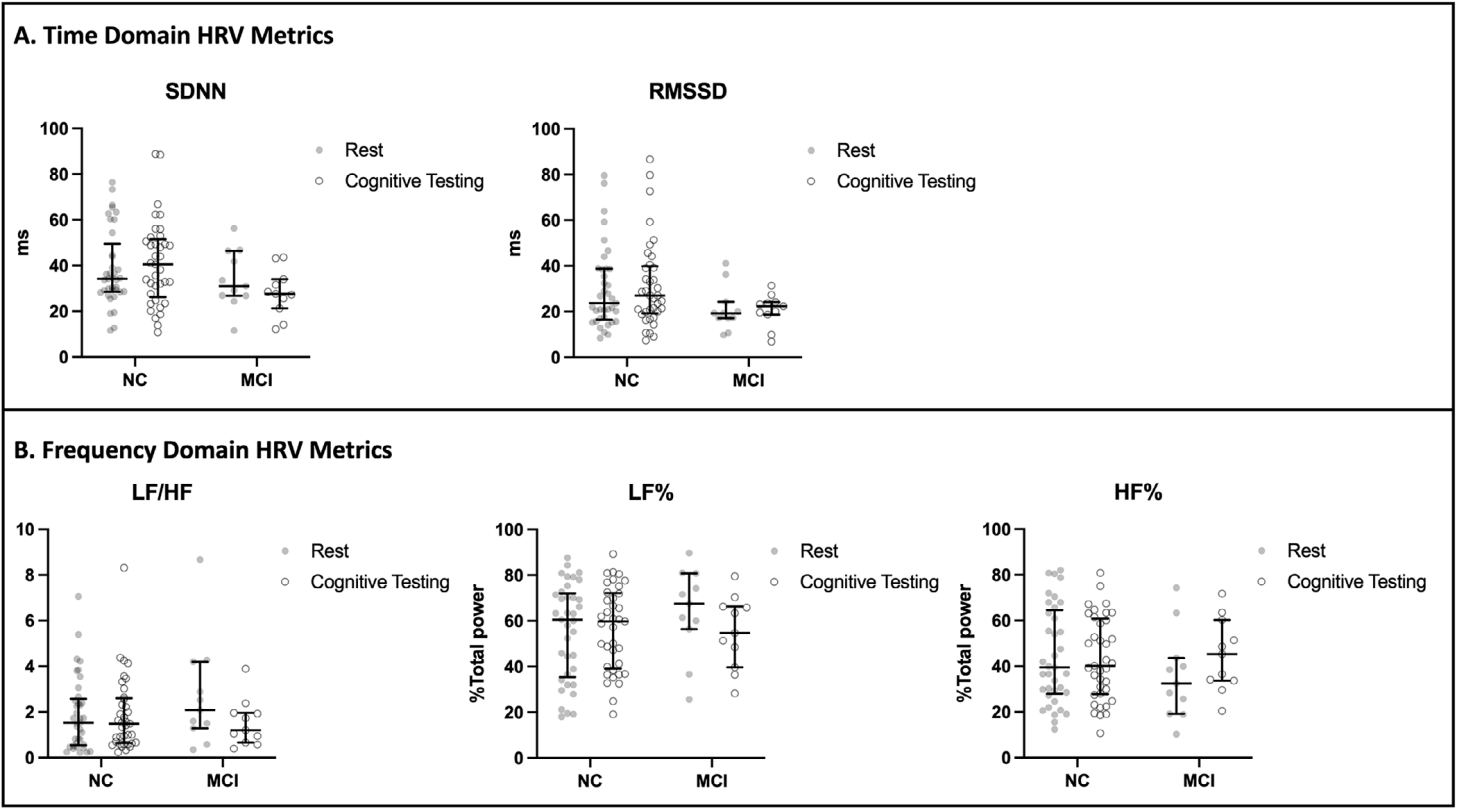# Heart rate variability during routine cognitive testing: preliminary analyses of time and frequency domain metrics

**DOI:** 10.1002/alz.092807

**Published:** 2025-01-09

**Authors:** James R. Bateman, Christopher L. Schaich, Hossam A Shaltout, Kathryn H Alphin, Samuel N. Lockhart, Timothy M. Hughes, Kristen A Lindquist, Suzanne Craft

**Affiliations:** ^1^ Wake Forest University School of Medicine, Winston‐Salem, NC USA; ^2^ The University of North Carolina, Chapel Hill, NC USA

## Abstract

**Background:**

Early autonomic function changes in Alzheimer’s disease (AD) may represent a biomarker for early affective changes in prodromal disease. We report preliminary differences in metrics of heart rate variability (HRV) before and during routine cognitive testing.

**Method:**

We enrolled 50 participants from the Wake Forest Alzheimer’s Disease Research Center to wear continuous ECG devices during their visit to assess time and frequency domain based metrics of HRV over 5 minutes at rest and during cognitive testing. We used linear mixed effects models adjusted for age, sex, race, education, and APOE genotype to estimate the effect of condition (rest vs. cognitive testing) on HRV by cognitive status.

**Result:**

The sample (n=50) included 39 (78%) individuals adjudicated as having normal cognition (NC), 11 (22%) had MCI, 72% women, 12% Black, 86% White, and 2% Asian. The average age was 73±7 years old, and average education was 15.5±2.6 years. There were no significant differences in time‐ or frequency‐based metrics of HRV between rest and cognitive testing in the total sample (Figure 1). MCI tended towards lower root mean square of successive differences (RMSSD; p=0.11) and higher low‐frequency/high‐frequency (LF/HF) ratio (p=0.062) at rest. RMSSD (p=0.05) but not LF/HF (p=0.99) remained lower during cognitive testing (Table 1, Figure 2).

**Conclusion:**

Preliminary findings from our ongoing study using gold‐standard continuous ECG‐tracing during routine cognitive testing suggest no differences in HRV during the rest and cognitive testing conditions among the total sample, but may preliminarily suggest a shift to higher sympathetic and lower parasympathetic tone in participants with MCI.